# Influence of the microbiota on epigenetics in colorectal cancer

**DOI:** 10.1093/nsr/nwy160

**Published:** 2018-12-22

**Authors:** Danfeng Sun, Yingxuan Chen, Jing-Yuan Fang

**Affiliations:** Division of Gastroenterology and Hepatology, Key Laboratory of Gastroenterology and Hepatology, Ministry of Health, State Key Laboratory for Oncogenes and Related Genes, Shanghai Institute of Digestive Disease, Renji Hospital, School of Medicine, Shanghai Jiao Tong University, Shanghai 200001, China

**Keywords:** colorectal cancer, gut microbiota, epigenetics, short-chain fatty acids

## Abstract

Colorectal cancer is one of the most common malignancies and is the second leading cause of cancer death worldwide. Generally, there are three categories of colorectal cancer development mechanism—genetic, epigenetic and aberrant immunological signaling pathways—all of which may be initiated by an imbalanced gut microbiota. Epigenetic modifications enable host cells to change gene expression without modifying the gene sequence. The microbiota can interact with the host genome dynamically through the interface presented by epigenetic modifications. In particular, bacterially derived short-chain fatty acids have been identified as one clear link in the interaction of the microbiota with host epigenetic pathways. This review discusses recent findings relating to the cross talk between the microbiota and epigenetic modifications in colorectal cancer.

## INTRODUCTION

Colorectal cancer is the second most common cancer in women and the third in men worldwide [1]. Multiple genetic mutations and epigenetic modifications contribute to the pathogenesis of colorectal cancer, which were first described in a milestone study by Fearon and Vogelstein [2]. However, the worldwide geographical variation in colorectal cancer incidence is remarkable [1]. Classical Japanese migration studies and numerous *in vitro* studies have all provided convincing evidence that environmental factors are responsible for the tumorigenesis of colorectal cancer, rather than genetic dysfunction.

Commensal microbes, present at different mucosal surfaces of the mammalian host, play an important role in processing environmental signals (e.g. diet). The gut microbiota comprises approximately 10^14^ bacterial cells that mostly exhibit commensalism with the host [3], and they are undoubtedly significant for host health and disease. Numerous studies in patients and experimental animals (mice) have linked the microbiota to colorectal tumorigenesis. Advances in sequencing and computational technology have facilitated the determination of the role of the gut microbiota in colorectal cancer. However, the precise mechanisms by which this process occurs remain poorly understood. It has been proposed that the microbiota contributes to carcinogenesis via three major routes: changing host cell proliferation or turnover, influencing host cell immune function, and metabolizing dietary factors and host-derived products [4,5]. In colorectal cancer, the microbiota promotes tumorigenesis through altered host–microbiota interactions and dysbiosis. Accordingly, germ-free (GF) status and wide-spectrum antibiotic treatment significantly reduced tumor numbers in chemical and genetic experimental models of colorectal carcinogenesis [6–9]. Studies showed that microbiota-derived metabolites participate in beta-oxidation and many other metabolic processes. In addition, accumulated data indicate that these metabolites can interact with epigenetic modifications in the host, thus enabling manipulation of the host chromatin’s state and functionality.

Epigenetic modifications are central mechanisms involved in directing transcriptional responses to environmental cues. Epigenetics has become an important area of research in the field of cancer biology, because of our increasing understanding of the specific epigenetic mechanisms involved in gene expression regulation. Epigenetic modifications are heritable, potentially reversible and regulate gene expression through DNA modification, histone modification and non-coding RNAs. A potentially significant interface provided by epigenetic machinery links the microbiota to a dynamic interaction with the host genome. Thus, understanding how epigenetic modifications are influenced by the intestinal microbiota could provide potential therapeutic targets to prevent and treat colorectal cancer. In this review, we discuss the mechanisms of epigenetic modulation in the development and progression of colorectal cancer, which may be influenced by the gut microbiota and microbial metabolites, and may therefore be amenable to prevention or therapeutic intervention.

## EPIGENETICS

Eukaryotic cells package their DNA around histone proteins, thus forming a higher-order structure called chromatin. The basic repeating units within chromatin are nucleosomes, which contain DNA wound around a histone octamer. Nucleosomes are linked by histone H1 and can perform further condensation of the chromatin structure [10]. In general, condensed chromatin (heterochromatin) is considered to repress gene expression as it physically limits the recruitment of the transcriptional complex to the DNA, while open chromatin (euchromatin) is more commonly considered to enable active gene expression [11]. Epigenetics broadly refers to the dynamic and reversible modification of a cell's transcriptional potential without changing the genetic sequence. Epigenetics encompasses ATP-dependent chromatin remodeling, and regulation by covalent nucleosomal modifications and non-coding RNAs [12].

Covalent nucleosomal modifications, as well as ATP-dependent remodeling enzymes, enable chromatin flexibility in response to endogenous and environmentally derived signals. Thus, various processes—such as DNA replication, repair, or transcription—can be regulated by the chromatin structure through local condensation or relaxation [13]. The most well-characterized covalent epigenetic modifications are DNA methylation and histone modifications, such as acetylation, methylation, phosphorylation, SUMOylation (SUMO, small ubiquitin-like modifier) and ubiquitination. These modifications, termed the ‘histone code’, regulate the recruitment of the transcriptional machinery and cofactors, and, thus, epigenetics is thought to be a central mechanism by which the environment influences mammalian transcriptional potential in health and disease states [14]. These epigenetic modifications are catalyzed and balanced by various modifying enzymes, such as DNA methyltransferases (DNMTs), histone acetyltransferases (HATs)/ histone deacetylases (HDACs) and histone methyltransferases (HMTs)/histone demethylases (HDMs). Recently, HDACs have been identified as targets of microbiota-derived metabolites and are therefore discussed in more detail below. Non-coding RNAs, including microRNAs (miRNAs), small interfering RNA and long non-coding RNA, also play important roles in epigenetics [15].

The study of gene mutations in colorectal cancer established our first understanding of the molecular alterations in colorectal cancer and has led to increasing numbers of studies of epigenetic alterations in cancer. The link between epigenetic modifications and cancer was first made in 1983 [16]. The epigenetic mechanisms that have a role in cancer development include: DNA methylation, histone modifications, miRNAs and non-coding RNAs, and nucleosome positioning [17]. Therefore, in this review, we discuss the impact of the gut microbiota on these epigenetic modifications during colorectal carcinogenesis.

## DIET AND GUT MICROBIOME METABOLITES

The most important environmental factor associated with colorectal cancer is diet. Epidemiological studies have demonstrated that the Western diet, rich in meat and fat, is a risk factor for colorectal cancer, whereas diets rich in fiber, particularly of cereals and whole grains, protect against colorectal cancer [18–20]. Complex carbohydrate fibers are the major undigested dietary residues that enter the colon and provide an energy source for the microbiota. High-fiber foods contain complex phytochemicals that can be metabolized by the gut microbes into short-chain fatty acids (SCFAs), including acetate, butyrate and propionate. Polyphenolic derivatives can interact with human intestinal epithelial cells and may modify epigenetic function to control gene expression [19]. Acetate is the most abundant SCFA, which is produced by most intestinal bacteria as a fermentation product. Propionate is mostly produced through the succinate pathway by Bacteroidetes and by some Firmicutes that belong to the Negativicutes class (such as *Phascolarctobacterium succinatutens*, *Dialister spp.* and *Veillonella spp.*) [21]. Butyrate is formed via the butyryl-CoA:acetate CoA-transferase route by some Firmicutes (including *Faecalibacterium prausnitzii*, *Roseburia spp.*, *Eubacterium rectale*, *Eu.hallii* and *Anaerostipes spp*.) [22–24]. Butyrate is preferentially used as an energy source by intestinal epithelial cells. Intracellular butyrate and propionate (but not acetate) can inhibit the activity of HDACs in gut epithelial cells and immune cells, promoting the hyperacetylation of histones and certain transcription factors that are involved in signal transduction, and thus playing a vital role in cancer development [25,26]. Fecal butyrate levels might be a biomarker of cancer risk, as well as cancer progression and severity [27]. Patients with advanced colorectal cancer have decreased butyrate producing bacteria and lower levels of SCFAs compared with healthy controls [28–30].

Compared with carbohydrate, the quantities of protein consumed in the diet are commonly lower, and its digestion and absorption by the small intestine is more efficient (>95%) [31]; therefore, the proteolytic fermentation quantities are smaller than those of saccharolytic fermentation. However, diets rich in meat can generate more inflammatory and carcinogenic metabolites via proteolytic fermentation, notably phenols, ammonia, branched-chain SCFAs, and other nitrogen-rich metabolites [32]. Hydrogen sulfide is produced by sulfate-reducing bacteria, such as *Desulfovibrio vulgaris*, in response to sulfur compounds derived from diets with high protein and fat contents. Experimental studies concluded that hydrogen sulfide is proinflammatory [33] and genotoxic at physiological concentrations [34]. Aromatic amino acids can also be released by protein fermentation, and have shown the ability to damage cellular structures and increase permeability in experimental studies [35]. High-fat diets generate bile acids, and primary bile acids can be converted to potentially carcinogenic secondary bile acids by gut bacteria [36]. The inflammatory and proliferative effects induced by the consumption of meat and bile acids can be prevented by the simultaneous consumption of resistant starch (RS) [37].

Recent studies have focused on the link between diet, the gut microbiota and colorectal cancer [29,38]. An imbalanced diet influences the structure and function of the gut microbiota, leading to increased levels of metabolites that can induce inflammation and proliferation, ultimately increasing the risk of colorectal cancer. In this review, we will focus on how these metabolites influence epigenetic modification during colorectal cancer initiation and progression.

## THE IMPACT OF GUT MICROBIOTA ON EPIGENETIC MODIFICATIONS

### miRNAs

miRNAs are small non-coding RNAs (18–25 nt) found in plants, animals and some viruses, which regulate the translation of target genes by inducing the degradation of mRNAs or inhibiting translation. Changes in miRNA expression have been observed in colorectal cancer. For example, the expression of miR-4478 and miR-1295b-3p in stool specimens of patients with early colorectal cancer (I, II) was significantly lower than that of the normal group [39].

A recent study found that fecal miRNAs could affect the composition of the gut microbiota, indicating that host cells can regulate the microbial community. miRNA mimics were synthesized and added to a culture of *Fusobacteriumnucleatum* and *Escherichiacoli*. The transcripts of bacterial genes and bacterial growth were significantly altered after miRNA treatment. The abundance of miRNAs was inversely proportional to the microbial abundance in mice. This suggested that microbes may take up miRNAs, which in turn may affect the microbes [40]. miRNA-21 and miRNA-200b are two carcinogenic miRNAs that are frequently upregulated in colorectal cancer cells. Compared with untreated cells, *Leuconostocmesenteroides* significantly reduced the expression levels of miRNA-21 and miRNA-200b in conditioned medium-treated HT-29 cells. These data suggested that *L. mesenteroides* could act as an anti-oncomiRNA in HT-29 cells [41].

Several miRNAs associated with colorectal cancer, such as mir-182, mir-503 and mir-17∼92, can regulate multiple genes and pathways. These miRNAs can promote carcinogenesis and disease progression. Metabolites from microbial sources can change the expression of host genes in the colon, including miRNAs. The levels of MiR-92a were seven times higher in sporadic human colon cancer tissue than in the adjacent normal colon. Butyrate-induced p57 expression could be inhibited by exogenous miR-92a, thus reversing the beneficial function of butyrate on the proliferation and apoptosis of colon cancer cells [28]. The lectin Fap2, produced by *Fusobacterium* fermentation, binds to glycans produced by colorectal cancer and attaches to tumor tissues. Interestingly, the glycan biosynthesis pathway is enriched in miRNA targets related to colorectal cancer-associated bacteria. The increased production of glycan might increase the replenishment of certain bacteria, such as *Fusobacterium*, in tumor sites. This result indicates a potential new mechanism whereby miRNAs can attract specific microbes to the tumor microenvironment by regulating glycan biosynthesis, thereby promoting tumorigenesis [42]. Yuan *et al*. identified 76 miRNAs from colorectal cancer tumor tissues and normal tissues, including oncogenic mir-503, mir-182 and mir-17∼92 clusters. These known oncogenic miRNAs were associated with the relative abundance of multiple microbial populations, including Proteobacteria, Firmicutes and Bacteroidetes. In tumor and normal tissues, there were differences in bacterial function associated with differentially expressed miRNAs. *Akkermansia* is associated with miRNAs related to colorectal cancer pathways, while *Roseburia*, *Fusobacterium* and *Providencia* are associated with miRNAs related to other cancer pathways. One of the main findings was that colorectal cancer-related bacteria were associated with miRNAs that regulate genes involved in microbial interactions. For example, these miRNAs were involved in regulating the production of glycan, which is vital to recruit pathogenic bacteria to the tumor [43].

## DNA METHYLATION

DNA methylation is an important epigenetic modification that is associated with tumor formation. Methylation can alter the activity of a DNA fragment without changing the sequence. DNA methylation of a promoter usually inhibits gene transcription. Genes encoding secreted frizzled-related proteins (SFRPs) frequently demonstrate promoter hypermethylation and transcriptional silencing in patients with colorectal cancer [44]. Probiotics such as *Fa.prausnitzii*, *Eu.rectale* and *Lactobacillus* were significantly reduced after azoxymethane (AOM)/dextran sulfate sodium (DSS) treatment, compared with those in the control. The numbers of pathogenic bacteria, including *Desulfovibrio* sp. and *Enterococcus* spp., were significantly increased after AOM/DSS treatment compared with those in the control. These events increased the activity of DNMTs, which silenced a portion of tumor suppressor genes, such as *SFRP2*, by methylation of its promoter [45]. An increased Firmicutes/Bacteroidetes ratio led to reduced inflammation and increased interleukin (IL)-6 gene expression, but also increased DNA damage and MutL homolog 1 (MLH1) methylation status, thus decreasing specific gene expression [46].

Mima *et al*. used a molecular pathological epidemiology database of 1069 patients with colorectal cancer to measure *F.nucleatum* DNA in the tumor tissue. They found that the amount of *F.nucleatum* DNA was associated with shorter colorectal cancer-specific survival [47]. Higher *F.nucleatum* DNA levels were associated with CIMP (CpG island methylator phenotype)-high and LINE-1 hypomethylation, and these changes in key tumor molecular characteristics were associated with clinical outcomes of colorectal cancer [47]. Another study demonstrated the influence of gut microbes on the epigenetic regulation of host genes. The researchers studied Toll-like receptor 2 (*TLR2*)-knockout mice for DNA methylation and gene expression in the colonic mucosa. Two genes involved in the immune process, *Anpep* (alanyl aminopeptidase, membrane) and *Ifit2* (interferon induced protein with tetratricopeptide repeats 2), were found in the colonic mucosa of *Tlr2^−/−^* mice, and methylation levels in their promoter regions were increased. Epigenomic and transcriptomic modifications are related to changes in the composition of mucosa microorganisms. Some microbial species, including members of the Firmicutes, differed markedly in the abundance between wild-type and *Tlr2^−/−^* animals. This suggested that the alteration of the mucosal microbial composition caused by the deficit of *Tlr2* could lead to the changes in epigenetic modifications, one of which affects the level of gene transcription [48].

In a comparison of the gut microbes in GF and conventional mice, the developmental establishment of intestinal DNA methylation patterns was significantly inhibited in the absence of gut microbes. This was not caused by low levels of DNMT1 activity. It is thought that methylation deficits are caused by a general reduction of one-carbon metabolites, whose syntheses rely on gut microbial products. Thus, bacteria play a more complex guiding role, rather than simply promoting methylation. To confirm the direct link between DNA methylation and intestinal flora, Yu *et al*. conducted fecal microbiota transplant (FMT) experiments to conventionalize GF mice. The reconstructed gut microbiota significantly increased DNA methylation at multiple CpG sites of 3′CpG islands [49].

## HISTONE MODIFICATION

Histone proteins are major components of chromatin, wrapping DNA into nucleosomes and folding it into higher-order structures. Histones can be modified by covalent post-translational modifications (PTMs), termed the ‘histone code’, which determine the repression or activation of gene expression [50]. Histone acetylation is catalyzed by specific HATs called lysine acetyltransferases, which transfer an acetyl group from acetyl coenzyme A (acetyl-CoA) to lysine resides [51]. Acetylation is primarily associated with gene activation, which increases DNA accessibility to transcription factors. HDACs have opposite function compared to HATs since they remove the acetyl (acyl) moiety from lysine residues. Histone methylation is also an important epigenetic process, which recruits certain transcription factors to the chromatin. Enzymes that catalyze epigenetic modifications are sensitive to endogenous metabolites [52]. For example, HDACs can be inhibited by butyrate and propionate, which are produced by gut microbes. A mechanism has been reported in which butyrate acts by binding to the Zn^2+^ in the catalytic site of HDACs, which is sensitive to the molecular structure [53]. To investigate whether intestinal bacteria and their metabolites influence host chromatin states, Krautkramer *et al*. examined histone modification states in GF, conventionally raised (ConvR) and conventionalized (ConvD) mice [54]. They investigated 55 unique and combinatorial acetylated and methylated histone PTM states in proximal colon, liver and white adipose tissue (WAT). Colonization of the microbiota induced critical increases in H4 acetylation in all three tissues, and this effect was even more significant in ConvD mice. H3 methylation patterns also changed after microbiota colonization. There was a significant increase in H3K27me3+K36un in all three tissues from the ConvD mice compared with that in their GF controls. Thus, acetylated and methylated chromatin states of the host tissue can be affected by the gut microbiota in a site-specific and combinatorial fashion. Their results strongly indicated that the gut microbiota is a driver of host tissue chromatin regulation. The host diet affects the composition and metabolism of the gut microbial community [55,56], and research has demonstrated that microbes regulate host chromatin states in a diet-dependent manner: The consumption of a ‘Western-type’ diet prevents the chromatin changes that are mediated by the gut microbiota that occur in a polysaccharide-rich diet.

The 18 known HDACs are classified into four groups based on their subcellular location and homology to yeast HDACs [57]. HDAC1, HDAC2 and HDAC3 are class I HDACs. Their expressions were originally characterized in intestinal epithelial cells (IECs), and they play an important role in intestinal development and cancer [58,59]. As an inhibitor of HDACs, butyrate can modulate histone acetylation, thereby regulating the transcriptional activity of several genes and decreasing the incidence of colorectal cancer. However, the mediators of this mechanism are poorly understood. In addition, HDAC expression in IECs coordinates intestinal homeostasis, which depends on commensal bacteria [60]. Specifically, the loss of IEC intrinsic HDAC3 (HDAC^ΔIEC^) resulted in increased H3K9 Ac levels, decreased antimicrobial defense genes, the loss of Paneth cells, impaired IEC function and alterations to intestinal microbe composition. In contrast to the inhibitory function of butyrate, HDAC^ΔIEC^ mice showed increased susceptibility to intestinal damage and inflammation. However, the generation of GF HDAC^ΔIEC^ mice revealed that Paneth cell homeostasis and intestinal barrier function were largely restored, indicating that HDAC3 coordinates commensal microbe-derived signals to maintain normal host–commensal relationships; however, the specific mechanisms remain to be determined, together with the precise mechanism of butyrate-mediated HDAC inhibition. Previous studies have indicated that butyrate regulates genes by modulating the promoter regions containing Sp1 sites or putative butyrate-responsive elements (BREs). For example, calretinin (CALB2), a member of the EF-hand family of Ca^2+^-binding proteins, has been found in most poorly differentiated colon carcinomas. Its expression in colon cancer cells is negatively regulated by butyrate through a BRE flanking the TATA box, indicating the chemopreventive activity of butyrate [61].

Although the precise mechanism remains unknown, butyrate has been considered to reduce colonic inflammation, which is a critical risk factor for colorectal cancer, and is also linked to aberrant epigenetics in colorectal carcinogenesis. Indigestible RSs are substrates for gut-microbial metabolism and have been shown to protect against intestinal inflammation. Liu *et al*. investigated the effects of RS type 4 (RS4)-derived butyrate on the epigenetic inhibition of proinflammatory genes, and found that RS4-fed mice had higher cecal butyrate and increased H3K27me3 in the promoter of nuclear factor-kappa-B1 (NF-κB1) in the colon tissue. *In vitro*, the H3K27me3-enrichment was negatively regulated with the regulation of NF-κB1 in sodium butyrate-treated human colon epithelial cells [62]. Free fatty acid receptor 2 (FFAR2, also named G protein-coupled receptor 43 (GPR43)) is activated by SCFAs such as butyrate, and regulates colonic inflammation. In the AOM/DSS and Apc^Min/+^/DSS mice models, Pan *et al*. showed that FFAR2 deficiency promoted colon adenoma development. FFAR2-deficient mice showed enhanced cAMP-protein kinase A (PKA)-cAMP responsive element binding protein (CREB) pathway activity that led to the overexpression of HDACs. H3K27me3 and H3K4me3 histone marks bind to the promoter regions of inflammation suppressors (e.g. *Sfrp1* (secreted frizzled-related protein 1), *Dkk3* (Dickkopf Wnt signaling pathway inhibitor 3) and *Socs1* (suppressor of cytokine signaling 1)), leading to downregulation of these genes in FFAR2-deficient mice. In addition, FFAR2 is vital for butyrate to suppress HDAC expression and induce the hypermethylation of inflammation suppressor genes, which suggested that FFAR2 is an epigenetic tumor suppressor [63]. Zheng *et al*. revealed that SCFAs, particularly butyrate, enhanced IEC barrier formation, and induced the epithelial anti-inflammatory IL-10 receptor alpha subunit (IL-10RA) mRNA and the IL-10RA protein by activating the signal transducer and activator of transcription 3 (STAT3) pathway and inhibiting HDACs. They also found that butyrate represses permeability-promoting claudin-2 tight junction protein expression via an IL-10RA-dependent mechanism [64]. Antimicrobial peptides (AMPs) are synthesized and secreted by immune and epithelial cells, and are essential for barrier defense, and reducing susceptibility to infection by modulating the expression of cytokines and chemokines; thus, they play a role in inflammatory diseases and cancer. Butyrate regulates the expression of AMPs in humans. Animal and human clinical studies of butyrate demonstrated that the increasing expression of AMPs in the colon protects against infection, thus establishing a mucosal barrier to prevent inflammation [65].

Paradoxically, butyrate can promote IEC proliferation under different situations. One molecular pathway depends on the butyrate concentration and the metabolic state of the cell. Near the base of colonic crypts, concentrations of butyrate (0.5 mM) are low and have no HDAC inhibitory effect. This butyrate is generally metabolized in the mitochondria to enhance cell proliferation through energetics. Higher butyrate concentrations (5 mM) are present near the lumen to stimulate colonocyte metabolism. Meanwhile, unmetabolized butyrate can spill into the nucleus and act as an HDAC inhibitor, stimulating differentiation and apoptosis, and inhibiting the proliferation of cancer cells [66,67]. This phenomenon is the so-called ‘butyrate paradox’. Thus, it is not surprising that certain studies [7] do not fit well with the concept that butyrate suppresses tumorigenesis.

## THE MICROBIOTA CONTROLS T CELL DIFFERENTIATION THROUGH EPIGENETIC REGULATION

The intestinal immune system plays a critical role in resisting invading pathogens; however, it can peacefully accommodate commensal microbes. This depends on two subtypes of T cells: T helper cells, which inhibit the gut bacteria, and regulatory T (Treg) cells that provide tolerance to the commensal microbes [68–71]. The commensal microbial community affects the delicate balance between pro- and anti-inflammatory mechanisms. Recent research shows that the microbiota controls T cell differentiation through epigenetic regulation, especially through histone modification. The majority of Treg cells expressing transcription factor forkhead box p3 (Foxp3) are generated in the thymus (tTreg); however, Tregs may also be generated at peripheral sites (pTregs) and play a vital role in repressing inflammatory responses in the gut [72]. In mice, an SCFA (butyrate), produced by commensal microbes during starch fermentation, promoted the extrathymic generation of Treg cells through its function as an HDAC inhibitor, which increased Foxp3 protein acetylation but not *FOXP3* messenger RNA levels [73]. Acetylation of Foxp3 enhanced its stability and function [74–76]. This regulation is dependent on the intronic enhancer, CNS1 (conserved-non-coding sequence 1), which is essential for extrathymic Treg cell differentiation [72,77]. Several studies have shown that individual commensal bacteria, from different phyla, are able to induce colonic pTreg cells [70,78–80]. Clostridia, a major class of commensal microbes, can induce colonic pTregs via their fermentation product, butyrate, which enhances histone H3 acetylation in the promoter and CNS1 of the *Foxp3* locus [78]. Other SCFAs, such as acetate and propionate, induce colonic Treg cells by activating GPR43 [81]. *Bacteroides*, such as *Bacteroidesthetaiotaomicron* and *B.fragilis*, induce specific colonic Tregs through polysaccharide A [79,82,83]. Interestingly, a divergent hypothesis regarding the interaction between pTregs and microbial metabolites has been raised recently. The CNS1 element was deleted in the *Foxp3* locus to selectively deplete pTregs, and metagenomics analysis was used to compare pTreg-deficient with pTreg-sufficient mice. The results showed that >80% of metabolites were significantly reduced in both the intestinal lumen and serum, which led the researchers to further identify differences in certain specific border-dwelling microbes [84]. Although the mechanism requires further elucidation, this correlation is very interesting.

## THE MICROBIOTA IN CANCER PREVENTION AND THERAPY

Most commercial drugs for colorectal cancer therapy are chemicals that were originally designed to suppress cancer cell growth or eliminate cancer cells. However, many of these drugs can cause serious side effects. Probiotics have been widely accepted as health supplements. Engineered beneficial microbes may show increasing antitumor properties. These microbes are commensal residents in the gut; therefore, they are much less likely to cause severe side effects than drugs [85]. *Lactobacillus bulgaricus* in yogurt, perhaps the most famous example of probiotics, can promote lactose digestion, enhance gut function and stimulate the intestine’s immune system. *Bifidobacteria* and *Streptococci* are also common in food and drink. FMTs can also be considered as probiotics and have been successfully applied to treat recurrent *Clostridiumdifficile* infections. Treatment of recurrent *C. difficile* infection with donor feces was more efficient than vancomycin [86]. By contrast, gavage with fecal samples from colorectal cancer patients could promote the development of intestinal cancer in both GF and conventional mice. Specifically, stools from patients with colorectal cancer increased the number of tumors, the grade of intestinal dysplasia and proliferation, levels of inflammatory cytokines, and proportions of Th1 and Th17 cells in the colon compared with feces of patients without colorectal cancer [87].

There is evidence that certain microbial populations are effectively in preventing or treating cancer, such as *Bifidobacterium* and *Bacteroides*. Cytotoxic T lymphocyte antigen-4 (CTLA-4) antibodies have been successfully used in cancer immunotherapy. The antitumor effects of CTLA-4 blockade depend on specific *Bacteroides* species. In mice and patients, T cells induced by *B. thetaiotaomicron* or *B. fragilis* were related to the immunotherapeutic efficacy of CTLA-4 blockade. Tumors in antibiotic-treated or GF mice did not respond to CTLA-4 blockade. This deficiency can be overcome by gavage with *B. fragilis*, by immunization with *B. fragilis* polysaccharides or by adoptive transfer of *B. fragilis-*induced T cells [88]. Sivan *et al*. compared the growth of melanomas in mice with different symbiotic floras and investigated the differences in spontaneous antitumor immunity. The differences were reduced after cohousing or fecal microbiota transplantation. They identified a link between *Bifidobacterium* and antitumor activity. Treatment of *Bifidobacterium* alone and the specific antibody of programmed cell death protein 1 ligand 1 (PD-L1) improved the control of tumors to some extent, and the combined treatment almost eliminated tumor growth. Thus, manipulation of the microbiota may modulate cancer immunotherapy [89].

Prebiotics are defined as digestible food ingredients that selectively enhance the growth and/or activity of certain intestinal flora, thereby benefiting health. Although all prebiotics contain fiber, not all fibers are prebiotics. Increased fiber consumption may reduce the risk of colorectal cancer. Insoluble fibers accelerate colon transport to reduce carcinogen exposure, while soluble fiber is fermented by gut microbes into SCFAs. In normal colon cells, butyrate is the main source of energy for homeostasis. In cancer cells, glucose is a major source of energy because of the Warburg effect. Butyrate is still transported into cancer cells through monocarboxylate transporters; however, there is no metabolism in the mitochondria, which leads to the accumulation of butyrate in the nucleus, which plays a role in the epigenetic regulation of cell proliferation and apoptosis as an HDAC inhibitor [90]. A recent powerful study recruited native African and African Americans to participate in a 2-week dietary intervention. Native Africans have a lower colorectal cancer rate (under 5 per 100 000) when switching from traditional high-fiber diets to low-fiber Western diets, while African Americans have a higher rate (>65 per 100 000) when switching from Western diets to high-fiber traditional diets. Changes in diet affect the intestinal flora, resulting in changes in metabolites, such as SCFAs, and intestinal biomarkers of cancer risk [91].

Chemotherapy failure is the major reason for recurrence and poor prognosis in colorectal cancer patients. In our recent study, we found that *F.nucleatum* was abundant in colorectal cancer tissues in patients with postchemotherapy recurrence, and *F. nucleatum* promoted colorectal cancer resistance to chemotherapy. Mechanistically, *F. nucleatum* targeted TLR4 and MYD88 innate immune signaling and specific microRNAs to activate the autophagy pathway, thereby altering colorectal cancer's chemotherapeutic response [92].

In the era of precision medicine, the integration of molecular pathology and epidemiology has led to the emergence of the transdisciplinary field of ‘molecular pathological epidemiology’ (MPE) [93]. The MPE approach can connect potential risk factors to the molecular pathology of a disease, and can contribute to precision medicine and precision prevention [94]. Colorectal cancer has been commonly studied in MPE research [93,95]. The gut microbiota plays an important role in malignant diseases; therefore, the field of microbiology can be readily integrated into the framework of MPE [96]. Gut microbiota influences the tumor response to immunotherapy and chemotherapy [92,97]; therefore, the analysis of the microbiota, diet, and colorectal carcinoma subtypes may be relevant to treatment decision-making.

## CONCLUSION AND PERSPECTIVES

**Figure 1.**
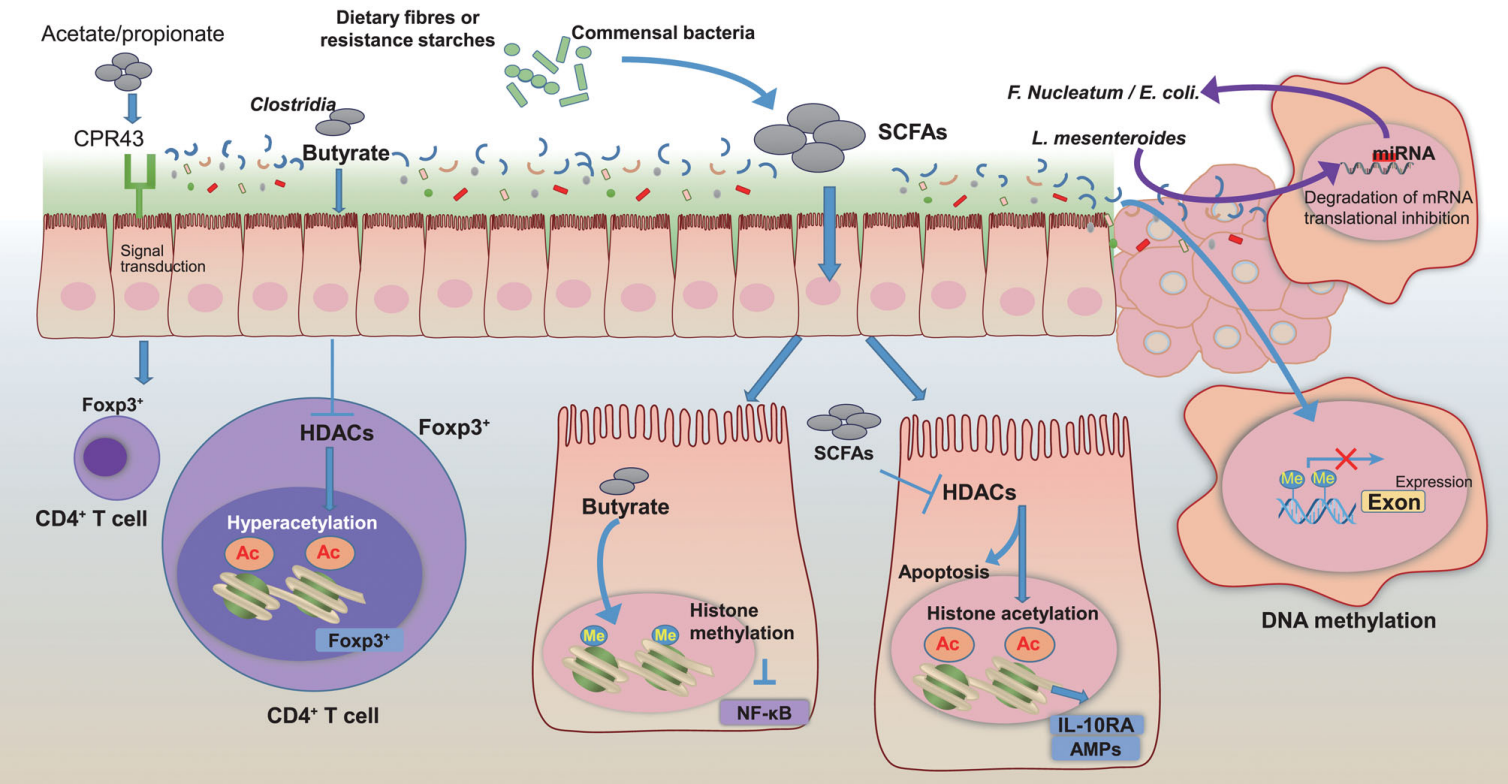
The gut microbiota influences epigenetic modifications in colorectal carcinogenesis. Commensal bacteria, such as Clostridia, can induce colonic pTregs via their fermentation product, butyrate, an HDAC inhibitor, which enhances histone H3 acetylation in the promoter and CNS1 of the *Foxp3* locus. Meanwhile, acetate and propionate promote the accumulation of colonic Treg cells by activating GPR43. Both induce colonic Foxp3^+^CD4^+^Treg cells, which have a key role in limiting inflammatory responses during carcinogenesis. Butyrate increases histone methylation in the promoter of NF-κB1, thus downregulates expression of NF-κB1. SCFAs induce the epithelial anti-inflammatory IL-10 receptor alpha subunit (IL-10RA) mRNA and antimicrobial peptides (AMPs) via their HDAC inhibitor functions. Thus, together with reduced NF-κB1, SCFAs inhibit colonic inflammation through epigenetic modification. In colon cancer cells, miRNAs alter the abundance of bacteria, such as *F. nucleatum* and *E. coli*. In contrast, bacteria (e.g. *L. mesenteroides*) can also influence the expression levels of miRNAs in colon cancer cells. In addition, commensal bacteria can influence gene expression by increasing DNA methylation at multiple CpG sites in colorectal cancer cells.

The gut microbiota plays a crucial role during colorectal carcinogenesis by either directly or indirectly affecting epigenetic modifications (Fig. 1). Mucosal inflammation, impaired barrier function and dysbiosis may result from dysregulation of the epigenetic cross talk between the host and microbiota. Recent studies have revealed new approaches to test host–microbiota interactions; however, our understanding of how epigenetic modifications participate in these interactions is just beginning. The identification of bacteria-derived metabolites that can induce and/or promote colorectal cancer development will be important future discoveries that will dramatically affect therapy. Microbes engineered to express specific genes or produce specific metabolites might be delivered to the gastrointestinal tract to treat or prevent cancer. Gut microbes also have the ability to modify the efficacy of chemotherapeutic drugs or immune checkpoint inhibitors. Integrating the gut microbiota into MPE research can contribute to precision medicine and prevention. Furthermore, by manipulating both the microbiota and the diet, we may achieve novel therapies that function via epigenetic modifications to fight against colorectal cancer development.
